# Supplementary material for: Downregulation of E-cadherin in pluripotent stem cells triggers partial EMT
Source: Sci Rep. 2021 Jan 21;11:2048. doi: 10.1038/s41598-021-81735-1 (PMC7820496; doi:10.1038/s41598-021-81735-1)
Supplement: Supplementary file 1 — Supplementary Information [file 41598_2021_81735_MOESM1_ESM.pdf]

Downregulation of E-cadherin in pluripotent stem cells triggers partial EMT

Aban C.E.[1]

Lombardi A.[1]

Neiman G.[1]

Biani M.C.[1]

La Greca A.[1]

Waisman A.[1]

Moro L.N.[1]

Sevlever G.[1]

Miriuka S.[1]

Luzzani C.[1,*]

[1] LIAN-CONICET, FLENI, Buenos Aires, Argentina

[*] Corresponding author: cluzzani@fleni.org.ar

Key words: Embryonic stem cells, E-cadherin, partial EMT

Supplemental Material

S1

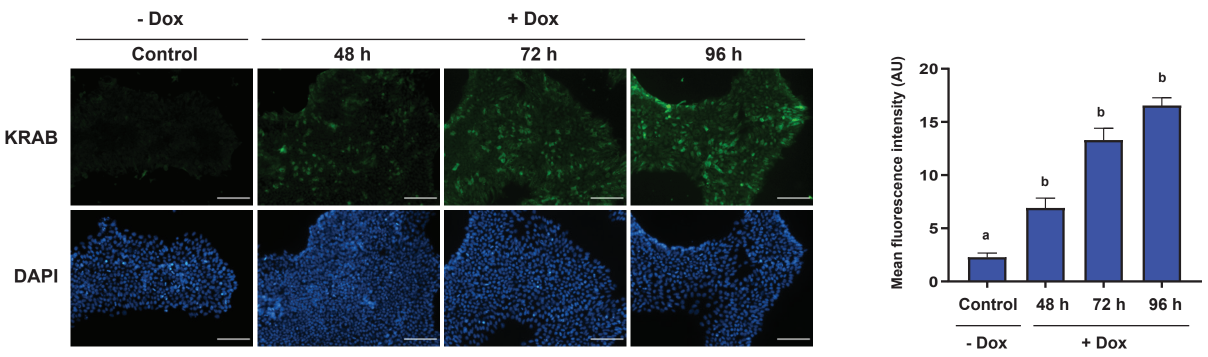

Figure S1. Expression of KRAB increased gradually after Dox incubation. Left panel: Immunofluorescence images of KRAB protein expression in cells incubated with Dox during 48, 72 and 96 h. Nuclei were stained with DAPI. Representative images of at least three experiments are shown. Scale bar 100 µm. Right panel: Quantification of the mean fluorescence intensity of three images from three independent experiments. Results are represented as mean ± SD (n=3). Different letters indicate significant differences of groups compared to control condition ($p<0.0001$) for ANOVA with post hoc Tukey.

S2

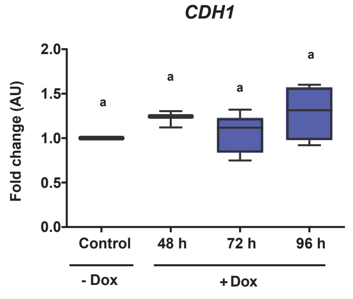

Figure S2. E-cadherin levels were not affected in parental HES3 cells after Dox incubation. Relative mRNA levels of CDH1 in parental HES3 wild type cells after incubation with Dox at different times assessed by qPCR. AU is the abbreviation for arbitrary units. Results are represented as mean ± SD (n=5). Different letters indicate significant differences of groups compared to control condition for ANOVA with post hoc Tukey.

S3

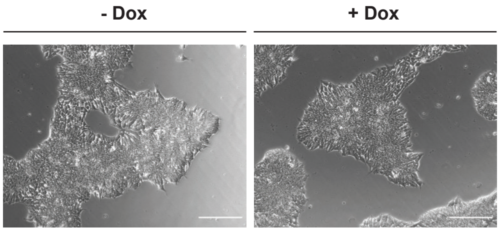

Figure S3. Cell morphology was not affected in parental HES3 cells incubated with Dox. Representative images of parental HES3 wild type cells incubated with (+Dox) or without Dox (-Dox) for 96h. Scale bar 200 µm.

S4

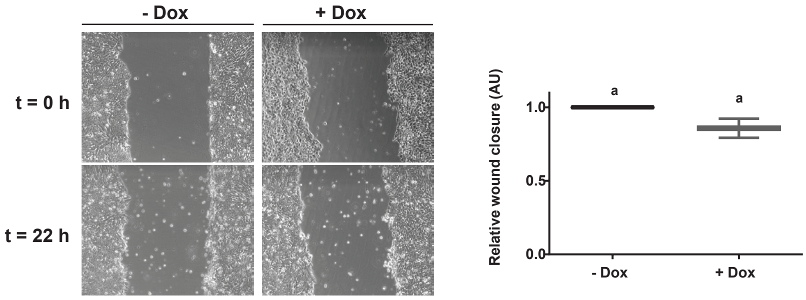

Figure S4. Cell migration was not enhanced in parental HES3 cells after Dox incubation. Wound healing assay was made after parental HES3 wild type cells were treated with or without Dox (+Dox and -Dox, respectively) (t=0h). Representative images of at least three experiments are shown. Images were taken at t=0 h and after 22 h of recovery (t=22h).

Table S1 - sgRNA sequences

Name	***Sequence***
sgRNA1 Coding	ACACCGCAGTTCCGACGCCACTGAGG
sgRNA1 Template	AAAACCTCAGTGGCGTCGGAACTGCG
sgRNA2 Coding	ACACCGCGCCGGGTGCGGTCGGGTCG
sgRNA2 Template	AAAACGACCCGACCGCACCCGGCGCG

Table S2 - Primers sequences

Gene	***Sequence***
CDH1	Forward AAAGGCCCATTTCCTAAAAACCT<br>Reverse TGCGTTCTCTATCCAGAGGCT
TJP1	Forward GGCAGCAAGAGATGGCAATA<br>Reverse ACGGTAGCCCGTTCAATCTCT
OCLN	Forward GTTGCGGCGAGCGGATTG<br>Reverse TGGACTTTCAAGAGGCCTGG
CLDN3	Forward ACGCGAGAAGAAGTACACGG<br>Reverse GTAGTCCTTGCGGTCGTAGC
GJA1	Forward GTGCCTGAACTTGCCTTTTC<br>Reverse CCCTCCAGCAGTTGAGTAGG
DSC2	Forward AACGGAGGTCAGGAGACCAT<br>Reverse TTTTTCACCAAGACGGGGCT
DSG2	Forward TTCGGGAGGGAGAGGATCTG<br>Reverse TCCTCTTGCATCCAAAGCGT
DSP	Forward GTGTCCTGGCACTACTGCAT<br>Reverse CTTCTGGGCATCGGTGAACT
NANOG	Forward AAAGGATCTTCACCTATGCC<br>Reverse GAAGGAAGAGGAGAGACAGT

OCT4	Forward	CTGGGTTGATCCTCGGACCT
	Reverse	CACAGAACTCATACGGCGGG
LIN28A	Forward	TCAGGCTTGGGTTCACACCATCAC
	Reverse	GGTTGCCCCAGAACCCTCAC
SNAI1	Forward	ATGCACATCCGAAGCCACA
	Reverse	GAGGGTCAGCGGGGACATC
SNAI2	Forward	TCAAGGACACATTAGAACTCAC
	Reverse	CTACACAGCAGCCAGATTC
ZEB1	Forward	TTACACCTTTGCATACAGAACCC
	Reverse	TTTACGATTACACCCAGACTGC
ZEB2	Forward	GGAGACGAGTCCAGCTAGTGT
	Reverse	CCACTCCACCCTCCCTTATTTC
TBX6	Forward	CAGCCTACCAGAACCCACAG
	Reverse	GTGTGTCTCCGCTCCCATAG
MIXL1	Forward	GGTACCCCGACATCCACTT
	Reverse	TGGAAGGATTTCCCACTCTG
LINC-ROR	Forward	CACTCCAGCTATGCAGACCA
	Reverse	CTGACCTGTTGACCCACCTT
MALAT1	Forward	TGTTCTGATCCCGCTGCTATT
	Reverse	ACGACTGCTTAAAACTGCAGAAA
CCND1 (cyclin D1)	Forward	GATCAAGTGTGACCCGGACT
	Reverse	TCCTCCTCCTCTTCCTCCTC
MYCBP (c-myc)	Forward	CCTGGCTCCCCTCCTGCCTCGA
	Reverse	GCTCCCTCTGCCTCTCGCTGGA
RPL7	Forward	AATGGCGAGGATGGCAAG
	Reverse	TGACGAAGGCGAAGAAGC
HPRT1	Forward	TGACACTGGCAAAACAATGCA
	Reverse	GGTCCTTTTCACCAGCAAGCT

NKX2.5	Forward CCCACGCCCTTCTCAGTCAA<br><br>Reverse GTAGGCCTCTGGCTTGAAGG
GATA4	Forward CATCAAGACGGAGCCTGGCC<br><br>Reverse TGACTGTCGGCCAAGACCAG
TUBB3	Forward TGGATTCGGTCCTGGATGTG<br><br>Reverse ACCTTGCTGATGAGCAACGT
